# [2-(4-Chloro­phen­yl)-5-phenyl­oxolan-3-yl](cyclo­penten­yl)methanone

**DOI:** 10.1107/S1600536811051993

**Published:** 2011-12-07

**Authors:** Jae Kyun Lee, Satish N. Chavre, Yong Seo Cho, Yeonhee Lee, Joo Hwan Cha

**Affiliations:** a Center for Neuro-Medicine, Korea Institute of Science & Technology, Hwarangro 14-gil, Seongbuk-gu, Seoul 136-791, Republic of Korea; b Advanced Analysis Center, Korea Institute of Science & Technology, Hwarangro 14-gil, Seongbuk-gu, Seoul 136-791, Republic of Korea

## Abstract

In the title compound, C_22_H_21_ClO_2_, the oxolane ring adopts a twisted conformation. The dihedral angles between the mean plane of the oxolane ring and the mean planes of the 4-chloro­phenyl, phenyl and cyclo­pentenyl rings are 71.81 (18), 76.9 (18) and 82.08 (18)°, respectively.

## Related literature

For general background to the Prins-type cyclization for the synthesis of oxolanes, see: Chavre *et al.* (2006 ▶, 2008 ▶); Cohen *et al.* (2001 ▶); Shin *et al.* (2005 ▶).
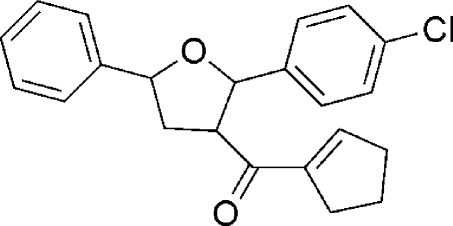


## Experimental

### 

#### Crystal data

C_22_H_21_ClO_2_
*M* *_r_* = 352.86
Monoclinic, 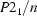

*a* = 5.7575 (6) Å
*b* = 11.3547 (12) Å
*c* = 28.554 (3) Å
β = 94.442 (8)°
*V* = 1861.1 (4) Å^3^
*Z* = 4
Cu *K*α radiation
μ = 1.90 mm^−1^
*T* = 296 K
0.50 × 0.20 × 0.20 mm

#### Data collection

Rigaku R-AXIS RAPID diffractometer
Absorption correction: multi-scan (*ABSCOR*; Rigaku, 1995 ▶) *T*_min_ = 0.301, *T*_max_ = 0.684
18544 measured reflections
3363 independent reflections
2012 reflections with *F* ^2^ > 2σ(*F* ^2^)
*R*_int_ = 0.073

#### Refinement

*R*[*F* ^2^ > 2σ(*F* ^2^)] = 0.051
*wR*(*F* ^2^) = 0.139
*S* = 1.05
3363 reflections
230 parameters
H atoms treated by a mixture of independent and constrained refinement
Δρ_max_ = 0.20 e Å^−3^
Δρ_min_ = −0.19 e Å^−3^

### 

Data collection: *RAPID-AUTO* (Rigaku, 2006 ▶); cell refinement: *RAPID-AUTO*; data reduction: *RAPID-AUTO*; program(s) used to solve structure: *SIR2008* in  *Il Milione* (Burla *et al.*, 2007 ▶); program(s) used to refine structure: *SHELXL97* (Sheldrick, 2008 ▶); molecular graphics: *CrystalStructure* (Rigaku, 2010 ▶); software used to prepare material for publication: *CrystalStructure*.

## Supplementary Material

Crystal structure: contains datablock(s) global, I. DOI: 10.1107/S1600536811051993/go2037sup1.cif

Structure factors: contains datablock(s) I. DOI: 10.1107/S1600536811051993/go2037Isup2.hkl

Supplementary material file. DOI: 10.1107/S1600536811051993/go2037Isup3.cml

Additional supplementary materials:  crystallographic information; 3D view; checkCIF report

## Figures and Tables

**Fig. 1.**
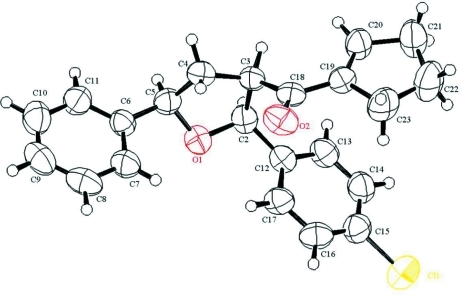
The structure of the title compound, with displacement ellipsoids drawn at the 50% probability level and H atoms with arbitrary radius.

## supplementary crystallographic information

### Comment

The oxolane moiety is an important heterocycle constituent in many bioactive natural products. New synthetic methodologies for 2,5-disubstituted oxolanes having an allenyl group, *via* Prins-type cyclization, has been subject of development since last decade (Shin *et al.*, 2005). Cohen and colleagues synthesized the polysubstituted oxolane by Prins-Pinacol reaction (Cohen *et al.*, 2001). 5-Exocyclic products, 2,3,5-trisustituted oxolanes, were synthesized from homopropargylic alcohols with alkynes and aldehydes (Chavre *et al.*, 2006, 2008).

In the molecule of Fig. 1, the oxolane ring adopts a twisted conformation on C5–O1. The dihedral angle between the chlorophenyl and cyclopentenyl rings is 31.03 (17)°.

### Experimental

To a stirred solution of 1-(4-hydroxy-4-phenylbut-1-ynyl)cyclopentanol (0.31 mmol) and 4-Chlorobenzaldehyde (0.37 mmol) in dry diethyl ether (3.0 ml) was added slowly TMSOTf (0.93 mmol) for 10 min and stirred for 1 h at -78°C. The mixture was allowed to warm to room temperature slowly for 3 h. The mixture was stirred at room temperature for additional 1–2 h until the completion of reaction. The reaction mixture was quenched with saturated aqueous NaHCO_3_ and diluted with 10 ml of diethyl ether. The organic solution was washed with water and brine, and the organic layer was dried over MgSO_4_, filtered, concentrated and purification by silica gel column chromatography to afford the title compound (54%) as a yellow crystal.

### Refinement

All hydrogen atoms were positioned geometrically and refined using a riding model with C—H = 0.93–0.98 Å and *U*iso(H) = 1.2 or 1.5 *U*eq(C). Rotating group model was applied for the methyl groups.

### Crystal data

**Table:**

C_22_H_21_ClO_2_	*F*(000) = 744.00
*M**_r_* = 352.86	*D*_x_ = 1.259 Mg m^−^^3^
Monoclinic, *P*2_1_/*n*	Cu *K*α radiation, λ = 1.54187 Å
Hall symbol: -P 2yn	Cell parameters from 12997 reflections
*a* = 5.7575 (6) Å	θ = 3.1–68.3°
*b* = 11.3547 (12) Å	µ = 1.90 mm^−^^1^
*c* = 28.554 (3) Å	*T* = 296 K
β = 94.442 (8)°	Needle, yellow
*V* = 1861.1 (4) Å^3^	0.50 × 0.20 × 0.20 mm
*Z* = 4	

### Data collection

**Table:**

Rigaku R-AXIS RAPID diffractometer	2012 reflections with *F*^2^ > 2σ(*F*^2^)
Detector resolution: 10.000 pixels mm^-1^	*R*_int_ = 0.073
ω scans	θ_max_ = 68.2°
Absorption correction: multi-scan (*ABSCOR*; Rigaku, 1995)	*h* = −6→6
*T*_min_ = 0.301, *T*_max_ = 0.684	*k* = −13→13
18544 measured reflections	*l* = −34→34
3363 independent reflections	

### Refinement

**Table:**

Refinement on *F*^2^	Secondary atom site location: difference Fourier map
*R*[*F*^2^ > 2σ(*F*^2^)] = 0.051	Hydrogen site location: inferred from neighbouring sites
*wR*(*F*^2^) = 0.139	H atoms treated by a mixture of independent and constrained refinement
*S* = 1.05	*w* = 1/[σ^2^(*F*_o_^2^) + (0.0568*P*)^2^ + 0.1808*P*] where *P* = (*F*_o_^2^ + 2*F*_c_^2^)/3
3363 reflections	(Δ/σ)_max_ = 0.001
230 parameters	Δρ_max_ = 0.20 e Å^−^^3^
0 restraints	Δρ_min_ = −0.19 e Å^−^^3^
Primary atom site location: structure-invariant direct methods	

### Special details

**Table:**

Geometry. All e.s.d.'s (except the e.s.d. in the dihedral angle between two l.s. planes) are estimated using the full covariance matrix. The cell e.s.d.'s are taken into account individually in the estimation of e.s.d.'s in distances, angles and torsion angles; correlations between e.s.d.'s in cell parameters are only used when they are defined by crystal symmetry. An approximate (isotropic) treatment of cell e.s.d.'s is used for estimating e.s.d.'s involving l.s. planes.
Refinement. Refinement was performed using all reflections. The weighted *R*-factor (*wR*) and goodness of fit (*S*) are based on *F*^2^. *R*-factor (gt) are based on *F*. The threshold expression of *F*^2^ > 2.0 σ(*F*^2^) is used only for calculating *R*-factor (gt).

### Fractional atomic coordinates and isotropic or equivalent isotropic displacement parameters (Å^2^)

**Table:**

	*x*	*y*	*z*	*U*_iso_*/*U*_eq_	
Cl(1)	0.34139 (17)	0.11812 (7)	0.53247 (3)	0.1065 (4)	
O(1)	0.2994 (3)	0.37307 (13)	0.74183 (5)	0.0597 (5)	
O(2)	0.6450 (3)	0.53421 (16)	0.66245 (6)	0.0763 (6)	
C(2)	0.1844 (5)	0.4198 (2)	0.70022 (8)	0.0574 (7)	
C(3)	0.2806 (5)	0.5488 (2)	0.69627 (8)	0.0561 (7)	
C(4)	0.4071 (5)	0.5703 (3)	0.74448 (8)	0.0700 (8)	
C(5)	0.3328 (5)	0.4685 (3)	0.77457 (8)	0.0602 (7)	
C(6)	0.5050 (5)	0.4344 (2)	0.81393 (8)	0.0541 (6)	
C(7)	0.7050 (5)	0.3738 (3)	0.80578 (10)	0.0682 (8)	
C(8)	0.8695 (5)	0.3488 (3)	0.84223 (12)	0.0802 (9)	
C(9)	0.8361 (6)	0.3839 (3)	0.88696 (11)	0.0805 (9)	
C(10)	0.6385 (6)	0.4435 (3)	0.89575 (9)	0.0795 (9)	
C(11)	0.4730 (5)	0.4682 (3)	0.85944 (9)	0.0668 (7)	
C(12)	0.2209 (5)	0.3392 (2)	0.65941 (8)	0.0526 (6)	
C(13)	0.0550 (5)	0.3341 (3)	0.62177 (9)	0.0628 (7)	
C(14)	0.0916 (5)	0.2667 (3)	0.58285 (9)	0.0713 (8)	
C(15)	0.2948 (6)	0.2045 (3)	0.58140 (9)	0.0680 (8)	
C(16)	0.4618 (5)	0.2066 (3)	0.61850 (10)	0.0697 (8)	
C(17)	0.4230 (5)	0.2737 (2)	0.65745 (9)	0.0632 (7)	
C(18)	0.4382 (5)	0.5586 (2)	0.65654 (8)	0.0549 (6)	
C(19)	0.3382 (5)	0.5935 (2)	0.60977 (8)	0.0523 (6)	
C(20)	0.1393 (5)	0.6481 (3)	0.59902 (9)	0.0667 (8)	
C(21)	0.1054 (6)	0.6755 (3)	0.54765 (9)	0.0881 (10)	
C(22)	0.3045 (6)	0.6163 (4)	0.52732 (10)	0.0999 (11)	
C(23)	0.4688 (5)	0.5744 (3)	0.56742 (9)	0.0829 (9)	
H(2)	0.0171	0.4240	0.7043	0.0689*	
H(3)	0.1505	0.6041	0.6912	0.0673*	
H(4A)	0.5746	0.5702	0.7426	0.0840*	
H(4B)	0.3612	0.6451	0.7573	0.0840*	
H(5)	0.1839	0.4877	0.7872	0.0723*	
H(7)	0.7295	0.3495	0.7755	0.0818*	
H(8)	1.0039	0.3077	0.8363	0.0963*	
H(9)	0.9478	0.3673	0.9113	0.0966*	
H(10)	0.6151	0.4674	0.9262	0.0954*	
H(11)	0.3380	0.5083	0.8657	0.0802*	
H(13)	−0.0827	0.3765	0.6227	0.0753*	
H(14)	−0.0211	0.2635	0.5577	0.0855*	
H(16)	0.5987	0.1636	0.6174	0.0836*	
H(17)	0.5345	0.2749	0.6828	0.0758*	
H(21A)	−0.0419	0.6444	0.5342	0.1057*	
H(21B)	0.1090	0.7598	0.5423	0.1057*	
H(22A)	0.3833	0.6710	0.5078	0.1199*	
H(22B)	0.2495	0.5501	0.5081	0.1199*	
H(23A)	0.5063	0.4918	0.5638	0.0994*	
H(23B)	0.6120	0.6198	0.5694	0.0994*	
H(20)	0.033 (5)	0.672 (3)	0.6215 (9)	0.086 (9)*	

### Atomic displacement parameters (Å^2^)

**Table:**

	*U*^11^	*U*^22^	*U*^33^	*U*^12^	*U*^13^	*U*^23^
Cl(1)	0.1507 (9)	0.0956 (7)	0.0744 (5)	0.0158 (6)	0.0156 (5)	−0.0156 (4)
O(1)	0.0594 (12)	0.0608 (11)	0.0580 (10)	−0.0031 (9)	−0.0002 (8)	0.0051 (8)
O(2)	0.0438 (12)	0.0850 (14)	0.1001 (14)	0.0143 (10)	0.0048 (10)	0.0100 (11)
C(2)	0.0435 (15)	0.0749 (17)	0.0541 (14)	0.0051 (13)	0.0051 (12)	0.0053 (12)
C(3)	0.0554 (16)	0.0592 (15)	0.0541 (14)	0.0195 (13)	0.0067 (12)	0.0033 (11)
C(4)	0.091 (2)	0.0526 (15)	0.0655 (16)	0.0113 (15)	−0.0014 (15)	−0.0010 (12)
C(5)	0.0540 (17)	0.0693 (17)	0.0580 (15)	0.0132 (13)	0.0085 (13)	−0.0031 (13)
C(6)	0.0498 (16)	0.0577 (14)	0.0546 (14)	0.0049 (12)	0.0032 (12)	0.0053 (11)
C(7)	0.0614 (19)	0.0715 (18)	0.0712 (17)	0.0147 (15)	0.0019 (14)	0.0040 (14)
C(8)	0.063 (2)	0.0660 (18)	0.109 (3)	0.0128 (15)	−0.0068 (18)	0.0108 (17)
C(9)	0.080 (3)	0.071 (2)	0.086 (3)	−0.0072 (17)	−0.0249 (18)	0.0174 (16)
C(10)	0.091 (3)	0.083 (2)	0.0624 (17)	−0.0093 (19)	−0.0075 (17)	−0.0003 (15)
C(11)	0.0663 (19)	0.0701 (17)	0.0639 (17)	0.0031 (14)	0.0038 (14)	−0.0034 (13)
C(12)	0.0423 (15)	0.0572 (14)	0.0580 (15)	−0.0024 (12)	0.0021 (12)	0.0057 (11)
C(13)	0.0521 (17)	0.0688 (17)	0.0670 (16)	0.0033 (14)	0.0018 (14)	0.0059 (13)
C(14)	0.077 (3)	0.0753 (19)	0.0592 (16)	0.0003 (16)	−0.0070 (15)	0.0029 (14)
C(15)	0.081 (3)	0.0638 (17)	0.0604 (16)	−0.0040 (16)	0.0129 (16)	0.0014 (13)
C(16)	0.0560 (18)	0.0653 (17)	0.089 (2)	0.0075 (14)	0.0120 (16)	−0.0044 (15)
C(17)	0.0489 (17)	0.0655 (16)	0.0741 (17)	0.0055 (13)	−0.0023 (13)	−0.0065 (13)
C(18)	0.0468 (16)	0.0497 (14)	0.0682 (16)	0.0050 (12)	0.0050 (13)	0.0004 (12)
C(19)	0.0449 (16)	0.0562 (14)	0.0567 (14)	−0.0011 (12)	0.0094 (12)	−0.0019 (11)
C(20)	0.0558 (18)	0.090 (2)	0.0554 (16)	0.0107 (16)	0.0102 (14)	0.0036 (14)
C(21)	0.073 (2)	0.132 (3)	0.0592 (17)	0.015 (2)	0.0067 (15)	0.0116 (17)
C(22)	0.093 (3)	0.146 (4)	0.0635 (19)	0.006 (3)	0.0223 (19)	−0.0039 (18)
C(23)	0.069 (2)	0.107 (3)	0.0762 (19)	0.0100 (18)	0.0269 (17)	−0.0053 (17)

### Geometric parameters (Å, °)

**Table:**

Cl(1)—C(15)	1.745 (3)	C(20)—C(21)	1.497 (4)
O(1)—C(2)	1.418 (3)	C(21)—C(22)	1.485 (5)
O(1)—C(5)	1.434 (3)	C(22)—C(23)	1.505 (4)
O(2)—C(18)	1.221 (3)	C(2)—H(2)	0.980
C(2)—C(3)	1.573 (4)	C(3)—H(3)	0.980
C(2)—C(12)	1.509 (4)	C(4)—H(4A)	0.970
C(3)—C(4)	1.526 (4)	C(4)—H(4B)	0.970
C(3)—C(18)	1.511 (4)	C(5)—H(5)	0.980
C(4)—C(5)	1.521 (4)	C(7)—H(7)	0.930
C(5)—C(6)	1.491 (4)	C(8)—H(8)	0.930
C(6)—C(7)	1.376 (4)	C(9)—H(9)	0.930
C(6)—C(11)	1.381 (4)	C(10)—H(10)	0.930
C(7)—C(8)	1.382 (4)	C(11)—H(11)	0.930
C(8)—C(9)	1.366 (5)	C(13)—H(13)	0.930
C(9)—C(10)	1.363 (5)	C(14)—H(14)	0.930
C(10)—C(11)	1.382 (4)	C(16)—H(16)	0.930
C(12)—C(13)	1.382 (4)	C(17)—H(17)	0.930
C(12)—C(17)	1.386 (4)	C(20)—H(20)	0.96 (3)
C(13)—C(14)	1.379 (4)	C(21)—H(21A)	0.970
C(14)—C(15)	1.370 (5)	C(21)—H(21B)	0.970
C(15)—C(16)	1.375 (4)	C(22)—H(22A)	0.970
C(16)—C(17)	1.380 (4)	C(22)—H(22B)	0.970
C(18)—C(19)	1.467 (4)	C(23)—H(23A)	0.970
C(19)—C(20)	1.318 (4)	C(23)—H(23B)	0.970
C(19)—C(23)	1.488 (4)		
			
O(2)···C(2)^i^	3.460 (3)	H(8)···H(4A)^iv^	3.5198
O(2)···C(8)^ii^	3.575 (4)	H(8)···H(4B)^iv^	3.3875
O(2)···C(13)^i^	3.537 (4)	H(8)···H(5)^i^	2.7276
C(2)···O(2)^iii^	3.460 (3)	H(8)···H(11)^i^	3.0564
C(8)···O(2)^iv^	3.575 (4)	H(8)···H(23B)^iv^	3.5412
C(8)···C(18)^iv^	3.475 (4)	H(8)···H(20)^iv^	3.2315
C(8)···C(19)^iv^	3.572 (4)	H(9)···Cl(1)^ii^	3.4433
C(11)···C(15)^v^	3.582 (4)	H(9)···Cl(1)^x^	3.5629
C(13)···O(2)^iii^	3.537 (4)	H(9)···C(19)^iv^	3.4151
C(15)···C(11)^vi^	3.582 (4)	H(9)···C(20)^iv^	3.4699
C(18)···C(8)^ii^	3.475 (4)	H(9)···C(21)^iv^	3.5022
C(19)···C(8)^ii^	3.572 (4)	H(9)···C(22)^iv^	3.5828
Cl(1)···H(9)^iv^	3.4433	H(9)···C(23)^iv^	3.4077
Cl(1)···H(9)^vii^	3.5629	H(9)···H(11)^i^	3.1235
Cl(1)···H(10)^vi^	3.4222	H(9)···H(16)^ii^	3.4677
Cl(1)···H(10)^vii^	3.3528	H(9)···H(21B)^iv^	3.0383
Cl(1)···H(11)^vi^	3.3964	H(9)···H(22A)^iv^	3.3010
Cl(1)···H(21B)^viii^	3.5133	H(9)···H(23B)^iv^	2.8899
Cl(1)···H(22A)^ix^	3.1389	H(10)···Cl(1)^v^	3.4222
O(1)···H(4B)^vi^	2.7496	H(10)···Cl(1)^x^	3.3528
O(1)···H(7)^iii^	3.4997	H(10)···C(15)^v^	3.5757
O(1)···H(8)^iii^	3.3806	H(10)···H(14)^v^	3.4412
O(2)···H(2)^i^	2.6806	H(10)···H(16)^ii^	3.0881
O(2)···H(3)^i^	3.0662	H(10)···H(21B)^vi^	2.8666
O(2)···H(8)^ii^	3.2230	H(11)···Cl(1)^v^	3.3964
O(2)···H(13)^i^	2.6883	H(11)···C(8)^iii^	3.2748
O(2)···H(20)^i^	3.03 (3)	H(11)···C(9)^iii^	3.3137
C(2)···H(4B)^vi^	3.3645	H(11)···C(14)^v^	3.2922
C(3)···H(8)^ii^	3.3504	H(11)···C(15)^v^	2.8298
C(5)···H(8)^iii^	3.2463	H(11)···C(16)^v^	2.8939
C(6)···H(8)^iii^	3.3292	H(11)···C(17)^v^	3.4096
C(6)···H(20)^vi^	3.52 (3)	H(11)···H(8)^iii^	3.0564
C(7)···H(2)^i^	3.5722	H(11)···H(9)^iii^	3.1235
C(7)···H(5)^i^	3.1280	H(11)···H(16)^v^	3.1385
C(7)···H(20)^vi^	3.44 (3)	H(13)···O(2)^iii^	2.6883
C(8)···H(5)^i^	2.9455	H(13)···C(16)^iii^	3.2505
C(8)···H(11)^i^	3.2748	H(13)···C(17)^iii^	3.2991
C(8)···H(20)^vi^	3.29 (3)	H(13)···H(16)^iii^	3.0320
C(9)···H(11)^i^	3.3137	H(13)···H(17)^iii^	3.1180
C(9)···H(16)^ii^	3.2006	H(13)···H(23A)^iii^	3.0864
C(9)···H(23B)^iv^	3.2533	H(13)···H(23B)^iii^	3.5534
C(9)···H(20)^vi^	3.20 (3)	H(14)···C(21)^viii^	3.0892
C(10)···H(16)^ii^	2.9601	H(14)···C(22)^viii^	3.1317
C(10)···H(21B)^vi^	3.1472	H(14)···H(10)^vi^	3.4412
C(10)···H(20)^vi^	3.26 (3)	H(14)···H(16)^iii^	3.0912
C(11)···H(8)^iii^	3.2826	H(14)···H(21A)^viii^	2.8734
C(11)···H(16)^ii^	3.3447	H(14)···H(21B)^viii^	2.8730
C(11)···H(20)^vi^	3.41 (3)	H(14)···H(22A)^viii^	2.7927
C(12)···H(4B)^vi^	3.3030	H(14)···H(22B)^viii^	3.0585
C(13)···H(16)^iii^	3.2583	H(16)···C(9)^iv^	3.2006
C(14)···H(11)^vi^	3.2922	H(16)···C(10)^iv^	2.9601
C(14)···H(16)^iii^	3.2907	H(16)···C(11)^iv^	3.3447
C(14)···H(21A)^viii^	3.4806	H(16)···C(13)^i^	3.2583
C(15)···H(10)^vi^	3.5757	H(16)···C(14)^i^	3.2907
C(15)···H(11)^vi^	2.8298	H(16)···H(9)^iv^	3.4677
C(15)···H(22A)^ix^	3.5571	H(16)···H(10)^iv^	3.0881
C(16)···H(11)^vi^	2.8939	H(16)···H(11)^vi^	3.1385
C(16)···H(13)^i^	3.2505	H(16)···H(13)^i^	3.0320
C(17)···H(4B)^vi^	3.3692	H(16)···H(14)^i^	3.0912
C(17)···H(11)^vi^	3.4096	H(17)···H(2)^i^	3.2711
C(17)···H(13)^i^	3.2991	H(17)···H(4B)^vi^	3.2998
C(18)···H(8)^ii^	2.8538	H(17)···H(13)^i^	3.1180
C(19)···H(8)^ii^	2.9837	H(21A)···C(14)^viii^	3.4806
C(19)···H(9)^ii^	3.4151	H(21A)···C(23)^iii^	3.1440
C(20)···H(8)^ii^	3.2142	H(21A)···H(14)^viii^	2.8734
C(20)···H(9)^ii^	3.4699	H(21A)···H(22A)^iii^	3.3502
C(20)···H(23B)^iii^	3.1048	H(21A)···H(22B)^viii^	2.7457
C(21)···H(9)^ii^	3.5022	H(21A)···H(23A)^iii^	3.2912
C(21)···H(14)^viii^	3.0892	H(21A)···H(23B)^iii^	2.3173
C(21)···H(22B)^viii^	3.5726	H(21B)···Cl(1)^viii^	3.5133
C(21)···H(23B)^iii^	3.0209	H(21B)···C(10)^v^	3.1472
C(22)···H(9)^ii^	3.5828	H(21B)···H(9)^ii^	3.0383
C(22)···H(14)^viii^	3.1317	H(21B)···H(10)^v^	2.8666
C(22)···H(22B)^ix^	3.4041	H(21B)···H(14)^viii^	2.8730
C(22)···H(23A)^ix^	3.1476	H(21B)···H(23B)^iii^	3.4145
C(23)···H(9)^ii^	3.4077	H(22A)···Cl(1)^ix^	3.1389
C(23)···H(21A)^i^	3.1440	H(22A)···C(15)^ix^	3.5571
C(23)···H(22B)^ix^	3.1328	H(22A)···H(9)^ii^	3.3010
H(2)···O(2)^iii^	2.6806	H(22A)···H(14)^viii^	2.7927
H(2)···C(7)^iii^	3.5722	H(22A)···H(21A)^i^	3.3502
H(2)···H(4A)^iii^	3.2970	H(22A)···H(22B)^ix^	3.3354
H(2)···H(4B)^vi^	3.4065	H(22A)···H(23A)^ix^	2.8646
H(2)···H(7)^iii^	2.8478	H(22B)···C(21)^viii^	3.5726
H(2)···H(17)^iii^	3.2711	H(22B)···C(22)^ix^	3.4041
H(3)···O(2)^iii^	3.0662	H(22B)···C(23)^ix^	3.1328
H(3)···H(8)^ii^	3.1877	H(22B)···H(14)^viii^	3.0585
H(4A)···H(2)^i^	3.2970	H(22B)···H(21A)^viii^	2.7457
H(4A)···H(7)^ii^	3.4183	H(22B)···H(22A)^ix^	3.3354
H(4A)···H(8)^ii^	3.5198	H(22B)···H(22B)^viii^	3.0914
H(4B)···O(1)^v^	2.7496	H(22B)···H(22B)^ix^	3.1672
H(4B)···C(2)^v^	3.3645	H(22B)···H(23A)^ix^	2.6197
H(4B)···C(12)^v^	3.3030	H(22B)···H(23B)^ix^	3.0847
H(4B)···C(17)^v^	3.3692	H(23A)···C(22)^ix^	3.1476
H(4B)···H(2)^v^	3.4065	H(23A)···H(13)^i^	3.0864
H(4B)···H(7)^ii^	3.4863	H(23A)···H(21A)^i^	3.2912
H(4B)···H(8)^ii^	3.3875	H(23A)···H(22A)^ix^	2.8646
H(4B)···H(17)^v^	3.2998	H(23A)···H(22B)^ix^	2.6197
H(5)···C(7)^iii^	3.1280	H(23B)···C(9)^ii^	3.2533
H(5)···C(8)^iii^	2.9455	H(23B)···C(20)^i^	3.1048
H(5)···H(7)^iii^	3.0467	H(23B)···C(21)^i^	3.0209
H(5)···H(8)^iii^	2.7276	H(23B)···H(8)^ii^	3.5412
H(7)···O(1)^i^	3.4997	H(23B)···H(9)^ii^	2.8899
H(7)···H(2)^i^	2.8478	H(23B)···H(13)^i^	3.5534
H(7)···H(4A)^iv^	3.4183	H(23B)···H(21A)^i^	2.3173
H(7)···H(4B)^iv^	3.4863	H(23B)···H(21B)^i^	3.4145
H(7)···H(5)^i^	3.0467	H(23B)···H(22B)^ix^	3.0847
H(8)···O(1)^i^	3.3806	H(23B)···H(20)^i^	2.8072
H(8)···O(2)^iv^	3.2230	H(20)···O(2)^iii^	3.03 (3)
H(8)···C(3)^iv^	3.3504	H(20)···C(6)^v^	3.52 (3)
H(8)···C(5)^i^	3.2463	H(20)···C(7)^v^	3.44 (3)
H(8)···C(6)^i^	3.3292	H(20)···C(8)^v^	3.29 (3)
H(8)···C(11)^i^	3.2826	H(20)···C(9)^v^	3.20 (3)
H(8)···C(18)^iv^	2.8538	H(20)···C(10)^v^	3.26 (3)
H(8)···C(19)^iv^	2.9837	H(20)···C(11)^v^	3.41 (3)
H(8)···C(20)^iv^	3.2142	H(20)···H(8)^ii^	3.2315
H(8)···H(3)^iv^	3.1877	H(20)···H(23B)^iii^	2.8072
			
C(2)—O(1)—C(5)	107.08 (17)	C(18)—C(3)—H(3)	109.756
O(1)—C(2)—C(3)	105.33 (18)	C(3)—C(4)—H(4A)	110.822
O(1)—C(2)—C(12)	109.62 (19)	C(3)—C(4)—H(4B)	110.830
C(3)—C(2)—C(12)	116.0 (2)	C(5)—C(4)—H(4A)	110.820
C(2)—C(3)—C(4)	103.30 (19)	C(5)—C(4)—H(4B)	110.820
C(2)—C(3)—C(18)	110.98 (19)	H(4A)—C(4)—H(4B)	108.881
C(4)—C(3)—C(18)	113.2 (2)	O(1)—C(5)—H(5)	109.307
C(3)—C(4)—C(5)	104.6 (2)	C(4)—C(5)—H(5)	109.305
O(1)—C(5)—C(4)	103.60 (18)	C(6)—C(5)—H(5)	109.316
O(1)—C(5)—C(6)	110.1 (2)	C(6)—C(7)—H(7)	119.692
C(4)—C(5)—C(6)	115.0 (2)	C(8)—C(7)—H(7)	119.701
C(5)—C(6)—C(7)	121.2 (3)	C(7)—C(8)—H(8)	119.783
C(5)—C(6)—C(11)	120.5 (3)	C(9)—C(8)—H(8)	119.795
C(7)—C(6)—C(11)	118.2 (3)	C(8)—C(9)—H(9)	120.091
C(6)—C(7)—C(8)	120.6 (3)	C(10)—C(9)—H(9)	120.106
C(7)—C(8)—C(9)	120.4 (3)	C(9)—C(10)—H(10)	120.016
C(8)—C(9)—C(10)	119.8 (3)	C(11)—C(10)—H(10)	120.013
C(9)—C(10)—C(11)	120.0 (3)	C(6)—C(11)—H(11)	119.505
C(6)—C(11)—C(10)	121.0 (3)	C(10)—C(11)—H(11)	119.501
C(2)—C(12)—C(13)	119.8 (3)	C(12)—C(13)—H(13)	119.632
C(2)—C(12)—C(17)	121.6 (2)	C(14)—C(13)—H(13)	119.645
C(13)—C(12)—C(17)	118.5 (3)	C(13)—C(14)—H(14)	120.183
C(12)—C(13)—C(14)	120.7 (3)	C(15)—C(14)—H(14)	120.201
C(13)—C(14)—C(15)	119.6 (3)	C(15)—C(16)—H(16)	120.539
Cl(1)—C(15)—C(14)	119.9 (2)	C(17)—C(16)—H(16)	120.562
Cl(1)—C(15)—C(16)	119.0 (3)	C(12)—C(17)—H(17)	119.414
C(14)—C(15)—C(16)	121.1 (3)	C(16)—C(17)—H(17)	119.421
C(15)—C(16)—C(17)	118.9 (3)	C(19)—C(20)—H(20)	124.5 (15)
C(12)—C(17)—C(16)	121.2 (3)	C(21)—C(20)—H(20)	123.6 (15)
O(2)—C(18)—C(3)	121.0 (2)	C(20)—C(21)—H(21A)	110.941
O(2)—C(18)—C(19)	119.8 (3)	C(20)—C(21)—H(21B)	110.944
C(3)—C(18)—C(19)	119.2 (3)	C(22)—C(21)—H(21A)	110.955
C(18)—C(19)—C(20)	127.7 (3)	C(22)—C(21)—H(21B)	110.945
C(18)—C(19)—C(23)	120.9 (3)	H(21A)—C(21)—H(21B)	108.957
C(20)—C(19)—C(23)	111.3 (3)	C(21)—C(22)—H(22A)	110.164
C(19)—C(20)—C(21)	111.8 (3)	C(21)—C(22)—H(22B)	110.161
C(20)—C(21)—C(22)	104.1 (3)	C(23)—C(22)—H(22A)	110.158
C(21)—C(22)—C(23)	107.7 (3)	C(23)—C(22)—H(22B)	110.167
C(19)—C(23)—C(22)	104.2 (3)	H(22A)—C(22)—H(22B)	108.463
O(1)—C(2)—H(2)	108.562	C(19)—C(23)—H(23A)	110.912
C(3)—C(2)—H(2)	108.556	C(19)—C(23)—H(23B)	110.920
C(12)—C(2)—H(2)	108.535	C(22)—C(23)—H(23A)	110.929
C(2)—C(3)—H(3)	109.737	C(22)—C(23)—H(23B)	110.927
C(4)—C(3)—H(3)	109.735	H(23A)—C(23)—H(23B)	108.947
			
C(2)—O(1)—C(5)—C(4)	−41.7 (2)	C(11)—C(6)—C(7)—C(8)	−0.6 (4)
C(2)—O(1)—C(5)—C(6)	−165.24 (17)	C(6)—C(7)—C(8)—C(9)	−0.0 (4)
C(5)—O(1)—C(2)—C(3)	34.0 (2)	C(7)—C(8)—C(9)—C(10)	0.4 (5)
C(5)—O(1)—C(2)—C(12)	159.47 (17)	C(8)—C(9)—C(10)—C(11)	−0.1 (5)
O(1)—C(2)—C(3)—C(4)	−12.5 (3)	C(9)—C(10)—C(11)—C(6)	−0.6 (5)
O(1)—C(2)—C(3)—C(18)	109.02 (18)	C(2)—C(12)—C(13)—C(14)	175.9 (2)
O(1)—C(2)—C(12)—C(13)	151.51 (18)	C(2)—C(12)—C(17)—C(16)	−175.4 (2)
O(1)—C(2)—C(12)—C(17)	−31.7 (3)	C(13)—C(12)—C(17)—C(16)	1.4 (4)
C(3)—C(2)—C(12)—C(13)	−89.4 (3)	C(17)—C(12)—C(13)—C(14)	−1.0 (4)
C(3)—C(2)—C(12)—C(17)	87.4 (3)	C(12)—C(13)—C(14)—C(15)	−0.2 (4)
C(12)—C(2)—C(3)—C(4)	−133.95 (19)	C(13)—C(14)—C(15)—Cl(1)	179.5 (2)
C(12)—C(2)—C(3)—C(18)	−12.4 (3)	C(13)—C(14)—C(15)—C(16)	1.1 (4)
C(2)—C(3)—C(4)—C(5)	−11.7 (3)	Cl(1)—C(15)—C(16)—C(17)	−179.07 (17)
C(2)—C(3)—C(18)—O(2)	−85.7 (3)	C(14)—C(15)—C(16)—C(17)	−0.6 (4)
C(2)—C(3)—C(18)—C(19)	91.9 (3)	C(15)—C(16)—C(17)—C(12)	−0.6 (4)
C(4)—C(3)—C(18)—O(2)	29.9 (3)	O(2)—C(18)—C(19)—C(20)	−162.7 (2)
C(4)—C(3)—C(18)—C(19)	−152.55 (18)	O(2)—C(18)—C(19)—C(23)	13.7 (4)
C(18)—C(3)—C(4)—C(5)	−131.75 (19)	C(3)—C(18)—C(19)—C(20)	19.7 (4)
C(3)—C(4)—C(5)—O(1)	31.9 (3)	C(3)—C(18)—C(19)—C(23)	−163.86 (18)
C(3)—C(4)—C(5)—C(6)	152.05 (19)	C(18)—C(19)—C(20)—C(21)	176.2 (2)
O(1)—C(5)—C(6)—C(7)	42.3 (3)	C(18)—C(19)—C(23)—C(22)	177.59 (19)
O(1)—C(5)—C(6)—C(11)	−140.95 (19)	C(20)—C(19)—C(23)—C(22)	−5.5 (3)
C(4)—C(5)—C(6)—C(7)	−74.2 (3)	C(23)—C(19)—C(20)—C(21)	−0.5 (3)
C(4)—C(5)—C(6)—C(11)	102.5 (3)	C(19)—C(20)—C(21)—C(22)	6.3 (4)
C(5)—C(6)—C(7)—C(8)	176.2 (2)	C(20)—C(21)—C(22)—C(23)	−9.4 (4)
C(5)—C(6)—C(11)—C(10)	−175.9 (2)	C(21)—C(22)—C(23)—C(19)	9.2 (4)
C(7)—C(6)—C(11)—C(10)	0.9 (4)		

Symmetry codes: (i) *x*+1, *y*, *z*; (ii) −*x*+3/2, *y*+1/2, −*z*+3/2; (iii) *x*−1, *y*, *z*; (iv) −*x*+3/2, *y*−1/2, −*z*+3/2; (v) −*x*+1/2, *y*+1/2, −*z*+3/2; (vi) −*x*+1/2, *y*−1/2, −*z*+3/2; (vii) *x*−1/2, −*y*+1/2, *z*−1/2; (viii) −*x*, −*y*+1, −*z*+1; (ix) −*x*+1, −*y*+1, −*z*+1; (x) *x*+1/2, −*y*+1/2, *z*+1/2.
